# Poor quality of life and functioning in bipolar disorder

**DOI:** 10.1186/s40345-017-0078-4

**Published:** 2017-03-27

**Authors:** Louisa G. Sylvia, Rebecca E. Montana, Thilo Deckersbach, Michael E. Thase, Maurcio Tohen, Noreen Reilly-Harrington, Melvin G. McInnis, James H. Kocsis, Charles Bowden, Joseph Calabrese, Keming Gao, Terence Ketter, Richard C. Shelton, Susan L. McElroy, Edward S. Friedman, Dustin J. Rabideau, Andrew A. Nierenberg

**Affiliations:** 1 Department of Psychiatry, Massachusetts General Hospital, 50 Staniford Street, Suite 580, Boston, MA 02114 USA; 2 Harvard Medical School, 25 Shattuck Street, Boston, MA 02115 USA; 3 Department of Psychiatry, University of Pennsylvania School of Medicine, Philadelphia, PA USA; 4 Department of Psychiatry and Behavioral Sciences, University of New Mexico, Health Sciences Center, Albuquerque, NM USA; 5 Department of Psychiatry, University of Michigan, Ann Arbor, MI USA; 6 Department of Psychiatry, Weill Cornell Medicine, New York City, NY USA; 7 Department of Psychiatry, University of Texas Health Science Center at San Antonio, San Antonio, TX USA; 8 Bipolar Disorders Research Center, University Hospital’s Case Medical Center, Case Western Reserve University, Cleveland, OH USA; 9 Department of Psychiatry and Behavioral Sciences, Stanford University School of Medicine, Stanford, CA USA; 10 Department of Psychiatry, University of Alabama Birmingham School of Medicine, Birmingham, AL USA; 11 Lindner Center of HOPE, Mason, OH USA; 12 Department of Psychiatry, University of Cincinnati College of Medicine, Cincinnati, OH USA; 13 University of Pittsburgh School of Medicine, Pittsburgh, PA USA; 14 Biostatistics Center, Massachusetts General Hospital, Boston, MA USA

**Keywords:** Bipolar disorder, Quality of life, Functioning, Social disadvantage

## Abstract

**Background:**

This study explores the association of demographic and clinical features with quality of life and functioning in individuals with bipolar disorder.

**Methods:**

Adult participants (*N* = 482) with bipolar I or II disorder were enrolled in a comparative effectiveness study across eleven study sites and completed baseline measures of medical and psychiatric history, current mood, quality of life, and functioning. Participants with at least mildly depressive or manic/hypomanic symptomatic severity were randomized to receive lithium or quetiapine in addition to adjunctive personalized treatment for 6 months.

**Results:**

Participants with more severe depressive and irritability symptoms had lower quality of life and higher functional impairment. All psychiatric comorbid conditions except substance use disorder were associated with worse quality of life. On average, females had lower quality of life than males. Patients who were married, living as married, divorced, or separated had *worse* functional impairment compared with patients who were single or never married. A composite score of social disadvantage was associated with worse functioning and marginally associated with worse quality of life. Symptom severity did not moderate the effect of social disadvantage on quality of life or functioning.

**Conclusions:**

Our findings highlight that depression, irritability, and psychiatric comorbid conditions negatively impact quality of life and functioning in bipolar disorder. The study suggests that individuals with social disadvantage are at risk for functional impairment.

*Trial Registration* This study is registered with ClinicalTrials.gov. Identification number: NCT01331304

## Background

Individuals with bipolar disorder frequently experience lower quality of life and worse functioning than the general population (Abraham et al. 2014; Sierra et al. 2005; Sylvia et al. 2013), even when not in a mood episode (Fulford et al. 2014; Gazalle et al. 2007; Shabani et al. 2013). Moreover, those who experience a lower quality of life exhibit higher inter-episode impulsivity and more cognitive impairment as well as residual depressive and psychotic symptoms (Depp et al. 2006; Victor et al. 2011).

Beyond psychiatric symptoms, other sources of stress could contribute to lower quality of life and functioning. One complex construct that might explain these deficits is social disadvantage. Social disadvantage is a composite measure composed of education level, employment status, income level, and occupational prestige (Duncan 1961). Bipolar disorder is associated with higher rates of unemployment and disability (Fulford et al. 2014; Sanchez-Moreno et al. 2009; Sylvia et al. 2013), and greater social disadvantage has been associated with higher levels of stress, higher mortality, and decreased access to healthcare (Adler and Newman 2002; Brennan et al. 2013; Mielck et al. 2014). Thus, it remains unclear as regards the association of social disadvantage with quality of life and functioning in bipolar disorder.

The purpose of this paper is to explore the determinants of quality of life and functioning, especially the construct of social disadvantage, in a representative cohort of bipolar patients who participated in a comparative effectiveness trial.

## Methods

### Participants

We enrolled 482 participants across eleven study sites for a comparative effectiveness trial [Bipolar CHOICE study—(Nierenberg et al. 2014)] that compared lithium with quetiapine in addition to adjunctive personalized treatment. The main inclusion criteria were being at least 18 years of age; having a primary diagnosis of bipolar I or II disorder; and being at least mildly symptomatic, either depressed, or manic/hypomanic. Participants were excluded if they were currently a psychiatric or medical inpatient, or had a history of failed treatment response to lithium or quetiapine, current lithium or quetiapine usage, and any contraindication to either medication (e.g., severe cardiovascular disease, renal disease, or pregnancy).

### Study design

The clinical and health outcomes initiative in comparative effectiveness for bipolar disorder study (Bipolar CHOICE) was a 6-month, parallel group, randomized controlled trial that compared the effectiveness of a second generation antipsychotic (quetiapine) and a classic mood stabilizer (lithium) (Nierenberg et al. 2014). Each group received adjunctive personalized treatments (APTs), or any other medications as needed, except the quetiapine group (QTP + APT) could not be prescribed lithium (Li + APT) or other antipsychotics and the lithium group could not be prescribed antipsychotics. APTs allow for greater generalizability and flexibility, as opposed to the strict monotherapy that patients typically receive in standard comparative effectiveness trials. APT was both evidence based and personalized to current symptoms, prior treatment history, and course of illness (Suppes et al. 2005).

Patients completed baseline measures assessing medical and psychiatric history as well as current mood, quality of life, and functioning and were then randomized to either Li + APT or QTP + APT. This report focuses on the baseline measures related to the quality of life.

### Measures

#### The mini international neuropsychiatric interview (Sheehan et al. 1997) (MINI 6.0)

The mini international neuropsychiatric interview is a clinician-administered, semistructured diagnostic interview that was designed specifically for clinical trials and epidemiological studies (Sheehan et al. 1997). The MINI was administered at the baseline visit to determine diagnoses, which correspond to DSM-IV-TR or ICD-10 diagnoses.

#### Quality of life, enjoyment, and satisfaction questionnaire (Endicott et al. 1993) (Q-LES-Q)

The Q-LES-Q is a self-report measure that assesses subjective quality of life (i.e., physical health, subjective feelings, leisure activities, and social relationships) over the previous week. Participants rate items on a scale from a 5-point scale that ranges from “very poor” to “very good.”

#### Longitudinal interval follow-up evaluation—range of impaired functioning tool (Leon et al. 2000) (LIFE-RIFT)

The LIFE-RIFT assesses the extent to which psychopathology has impacted current functioning in work, household chores, interpersonal relationships with partner, family, and friends, recreational activities, and life satisfaction. A reliable and valid measure of functioning, the LIFE-RIFT, assesses impairment in four areas—work (employment, household, school), interpersonal relationships (spouse, child, other relatives, friends), satisfaction, and recreation.

#### Social disadvantage

Social disadvantage was defined as the sum of three dichotomous variables: household income (1 if less than $25,000, 0 if $25,000 or more), education status (1 if highest level was high school or less, 0 if at least some college), and employment status (1 if unemployed/disabled, 0 if employed/student/retired). We omitted occupational prestige in the study’s definition of social disadvantage as it was not assessed given the subjectivity of this component and that prestige likely varies for this clinical population (i.e., based on high rates of disability, any job could, and probably should, be prestigious for an individual with bipolar disorder) (Oakes and Rossi 2003). It has been widely noted that low socioeconomic status is correlated with psychiatric illness and higher rates of disability (Hirschfeld et al. 2003; Lorant et al. 2003).

#### Bipolar inventory of symptoms scale (Bowden et al. 2007)

The BISS is a clinician-administered, structured interview that was originally developed with a focus on assessing bipolar symptoms in an outpatient sample. Items are assessed on a 5-point Likert scale, 0 indicating not at all to 4 indicating severe. The BISS measures the domains of mania, depression, irritability, anxiety, and psychosis (Thompson et al. 2010). BISS domain scores were re-scaled to range from 0 to 40.

### Statistical analysis

Descriptive data at baseline were reported as frequencies or mean with standard deviation. Linear regression models were fit to explore whether baseline quality of life (Q-LES-Q) and functioning (LIFE-RIFT) were associated with baseline demographics, clinical severity, social disadvantage, and current psychiatric comorbid conditions. The composite score of social disadvantage (calculated based on household income, education level, and employment status) ranged from 0 to 3, corresponding, respectively, to no, mild, moderate, and severe disadvantage. Variables (excluding comorbid conditions) were considered for inclusion in the multivariate linear regression model if univariate screening *p* value was less than 0.25 and were ultimately included in the model if the adjusted *p* value was less than 0.05.

To explore whether clinical symptom severity (BISS total) moderated the effect of social disadvantage on quality of life and functioning, we fit linear regression models with main effects for disadvantage and clinical severity as well as their interaction. If the interaction was significant, we determined there to be evidence of moderation. For these moderator analyses, we conducted a sensitivity analysis separating social disadvantage into each of its three components (rather than using the categorical composite score) to determine whether our conclusions were sensitive to this composite definition. Statistical analyses were performed using SAS 9.4 (Cary, NC, USA) and R version 3.2.1 (www.r-project.org).

## Results

At baseline, the mean quality of life (Q-LES-Q) score was 44.3 (SD = 17.8), and the mean functioning (LIFE-RIFT) score was 14.2 (SD = 3.4). The baseline associations of quality of life and functioning with demographics, clinical severity, current psychiatric comorbid conditions, and social disadvantage are reported in Table 1. On average, females had lower quality of life than males. Patients who were married, living as married, divorced, or separated had worse functional impairment (higher scores on LIFE-RIFT) compared with patients who were single or never married. Age, ethnicity, and race were not significantly related to quality of life or functioning.

**Table 1:** Baseline correlations of quality of life and functioning with demographics, clinical severity, social disadvantage, and current psychiatric comorbidities

Variable	% (n) or mean + SD	Q-LES-Q		LIFE-RIFT	
		Coefficient	*p* value	Coefficient	*p* value
Demographics					
Age	38.9 + 12.1	0.03	0.65	0.01	0.34
Female (REF: male)	59% (283)	−4.6	*0.006*	0.5	0.10
Hispanic/Latino (REF: not)	11% (53)	2.1	0.43	0.6	0.21
Race			0.94		0.83
White	72% (348)	REF		REF	
Black	20% (96)	−1.1		0.2	
Asian	3% (16)	0.7		0.1	
Native American	<1% (1)	5.6		0.9	
Other	4% (21)	2.2		0.9	
Marital status			0.66		0.05
Single/never married	47% (225)	REF		REF	
Married/living as married	31% (150)	−2.1		0.9	
Divorced/separated	21% (100)	−1.3		0.8	
Widowed	1% (7)	2.3		0.2	
Clinical symptom severity					
BISS total	56.1 + 18.8	−0.46	*<0.001*	0.09	*<0.001*
BISS depression	17.5 + 7.3	−1.64	*<0.001*	0.29	*<0.001*
BISS mania	9.2 + 6.4	0.23	0.08	−0.03	0.29
BISS anxiety	15.9 + 8.5	−0.92	*<0.001*	0.17	*<0.001*
BISS irritability	16.8 + 8.5	−0.65	*<0.001*	0.15	*<0.001*
BISS psychosis	2.8 + 4.5	−0.48	*0.007*	0.15	*<0.001*
Social disadvantage					
Household income <$25 K (REF: ≥25 K)	52% (249)	−1.7	0.30	0.6	0.06
High school diploma or less (REF: at least some college)	25% (122)	−2.0	0.29	0.8	*0.03*
Unemployed/disabled (REF: employed/student/retired)	51% (244)	−3.1	0.06	0.7	*0.03*
Social disadvantage			0.07		*0.03*
No disadvantage	28% (133)	REF		REF	
Mild	30% (142)	−1.1		0.6	
Moderate	29% (138)	−5.3		0.8	
Severe	13% (65)	−2.2		1.4	
Current psychiatric comorbidity					
Generalized anxiety disorder	22% (107)	−5.9	*0.002*	0.6	0.11
Substance use disorder	23% (109)	−0.6	0.76	0.2	0.49
Obsessive–compulsive disorder	11% (51)	−4.9	*0.02*	0.9	0.08
Panic disorder	23% (112)	−8.7	*<0.001*	0.8	*0.03*
Agoraphobia	37% (176)	−5.8	*0.001*	0.9	*0.003*
Social disorder	25% (119)	−5.3	*0.005*	0.8	*0.03*
ADHD	34% (161)	−4.2	*0.02*	1.0	*0.002*
Any anxiety disorder^a^	58% (277)	−8.9	*<0.001*	1.2	*<0.001*

*Q-LES-Q* quality of life enjoyment and satisfaction questionnaire, *LIFE-RIFT* longitudinal interval follow-up evaluation-range of impaired functioning tool, *REF* reference level, *BISS* bipolar inventory of symptoms scale, *ADHD* attention deficit hyperactivity disorder. Coefficients and *p* values based on linear regression models. Coefficients represent the change in Q-LES-Q/LIFE-RIFT per 1-point increase for continuous variables and change from reference level for categorical variables

^a^Includes patients with any of the following current diagnoses (based on MINI): panic disorder, agoraphobia, social phobia, and generalized anxiety disorder

**Fig. 1:**
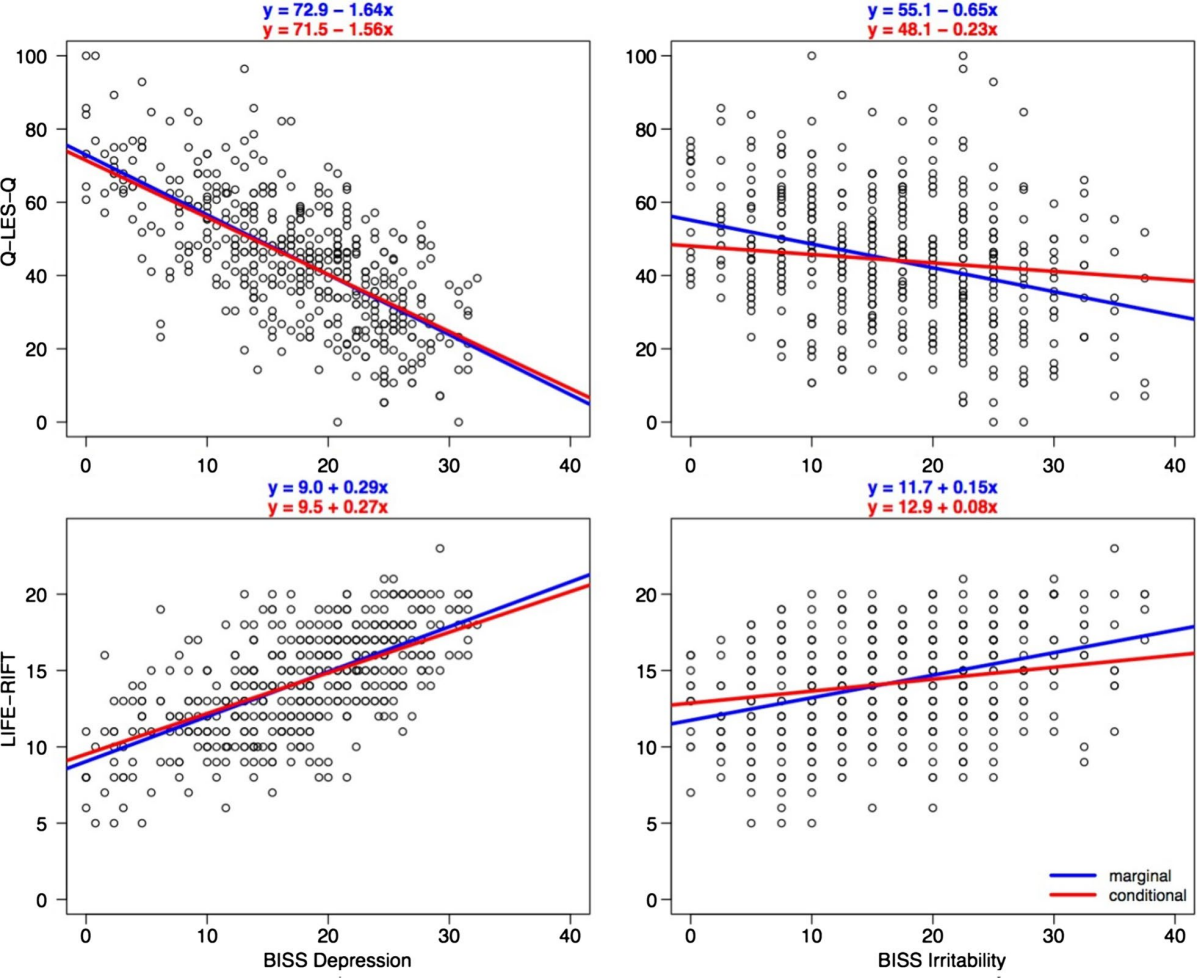
Marginal and conditional effects of depression and irritability on quality of life and functioning. In this figure, we display the marginal (unadjusted) and conditional (adjusted for other covariate in model) effects of depressive and irritability symptom severity on quality of life and functioning. In both cases, the marginal and conditional effects of BISS depression were similar, while the effect of BISS irritability decreased when adjusting for BISS depression

Participants with greater clinical symptom severity measured with BISS (total, depression, anxiety, irritability, and psychosis) had lower quality of life and higher functional impairment (Table 1). Patients with greater manic severity reported marginally better quality of life. No association was found between severity of manic symptoms and functioning. Only BISS depression and BISS irritability remained significant in both multivariate models (Q-LES-Q and LIFE-RIFT). We also found that although most of the BISS symptom domains (anxiety, irritability, depression, and psychosis) were associated with social disadvantage, mania was not (Table 2). In Fig. 1, we display the marginal (unadjusted) and conditional (adjusted for other covariate in model) effects of depressive and irritability symptom severity on quality of life and functioning. In both cases, the marginal and conditional effects of BISS depression were similar, while the effect of BISS irritability decreased when adjusting for BISS depression.

**Table 2:** Association between BISS symptom domains and social disadvantage

	Social disadvantage score				Overall
	0-REF	1	2	3	*p* value
BISS domain score					
Depression	16.3 + 7.1	16.7 + 7.1	*18.6* *+* *7.7* ^*a*^	*19.3* *+* *7.0* ^*a*^	0.006
Mania	8.13 + 5.9	9.2 + 6.8	9.9 + 6.9	9.4 + 4.8	0.15
Anxiety	13.8 + 8.0	15.6 + 8.3	*17.0* *+* *9.0* ^*a*^	*18.3* *+* *8.4* ^*a*^	0.001
Irritability	15.9 + 8.3	15.2 + 8.3	17.7 + 8.9	*20* *+* *7.9* ^*a*^	0.0006
Psychosis	1.7 + 3.2	2.6 + 4.3	*3.5* *+* *5.4* ^*a*^	*4.0* *+* *4.6* ^*a*^	0.0006

*REF* reference level

^a^Pairwise comparison with 0-REF is significant (*p* < 0.05)

Social disadvantage was significantly associated with worse functioning and marginally associated with worse quality of life (*p* = 0.07) (Table 1). Each component (income, education, and employment) was at least marginally associated with functioning, such that patients with lower income, lower education, and unemployed/disabled had worse functioning (all *p* < 0.07). Those who were unemployed or disabled were marginally associated with lower quality of life (*p* = 0.06), whereas neither income nor education was significantly associated with quality of life.

All psychiatric comorbid conditions, except substance-use disorder, were significantly associated with worse quality of life (Table 1). Panic disorder, agoraphobia, social disorder, ADHD, and any anxiety disorder were also associated with worse functional impairment. The total number of psychiatric comorbid conditions was associated with quality of life and functioning, such that for each additional comorbid condition, the mean quality of life decreased by 3.8 points and the mean functional impairment increased by 0.7 points (both *p* < 0.001). We found no evidence to suggest that clinical symptom severity moderated the effect of social disadvantage on quality of life and functioning (both *p* > 0.05).

## Discussion

Our results are consistent with earlier reports that show that individuals with bipolar disorder often experience overall poor quality of life and life functioning (Abraham et al. 2014; Sierra et al. 2005; Sylvia et al. 2013). We also found that females had lower quality of life than males. Patients who were married, living as married, divorced, or separated had worse functional impairment compared with patients who were single or never married. All psychiatric comorbid conditions, except substance-use disorder, were associated with worse quality of life. Panic disorder, agoraphobia, social disorder, ADHD, and any anxiety disorder were also associated with worse functional impairment. These findings are consistent with previous studies examining comorbid conditions and functioning in bipolar disorder (Mendlowicz and Stein 2000; Otto et al. 2006; Rapaport et al. 2005; Simon et al. 2004).

Social disadvantage was significantly associated with worse functioning and symptoms, but unexpectedly only marginally associated with quality of life. The multivariate models suggested that depression and irritability together were the strongest indicators of poor quality of life and functioning in individuals with bipolar disorder. Consistent with prior studies (Boylan et al. 2004; McElroy et al. 2001; Simon et al. 2004), our findings suggest that current symptom severity and psychiatric comorbid conditions negatively impact quality of life and functioning in bipolar disorder.

These results support findings in other clinical populations that social disadvantage can have a negative effect on the overall functioning of individuals with bipolar disorder (Bowie et al. 2010; Huxley and Baldessarini, 2007; Judd and Akiskal 2003). Social disadvantage has been associated with higher levels of stress, greater likelihood of depression, and higher rates of disability (Lorant et al. 2003). Given the frequency of depression and irritability as well as severity of these symptoms in this clinical population, it is perhaps not surprising that these symptoms were primarily associated with social disadvantage among individuals with bipolar disorder; however, we expected that mania would correlate with this outcome given the degree to which these symptoms can disrupt one’s functioning (e.g., ability to work, obtain high incomes). It is likely that we did not find this correlation as most participants were depressed (opposed to manic) throughout the duration of the study (Tohen et al. 2015) (see Table 1).

Interestingly, while patients with greater clinical severity (i.e., total, depression, anxiety, irritability) had lower quality of life, patients with greater manic severity reported marginally better quality of life. No association was found between manic severity and functioning, consistent with the literature (Rosa et al. 2010), and mania was not associated with social disadvantage. These findings could be explained by the use of a self-report measure of quality of life as this relies on the insight and perception of the individual, which are often altered during this mood state with grandiosity and expansiveness. This study is unique in its analysis of the association of mood symptoms with social disadvantage in a large sample of bipolar I and bipolar II patients; however, it should be noted that education and employment (two components of social disadvantage) are potentially overlapping constructs of quality of life and functioning. Further, the limited inclusion and exclusion criteria may have enhanced generalizability of our findings. However, these participants were recruited for a study at academic medical centers, and thus, replication in community settings is warranted.

## Conclusions

Individuals with bipolar disorder commonly report poor quality of life and have difficulty functioning. This study also sought to explore those who might be at greater risk for poor outcomes by examining the impact of a composite variable of several, overlapping demographic aspects, or social disadvantage. It appears that depression and irritability (but not mania) worsen quality of life and functioning in this clinical population. Overall, these data suggest that better interventions for individuals with social disadvantage, depression, and irritability are needed as they may be at greater risk for improvement in quality of life and functioning.
